# Predictors of health decline in older adults with pneumonia: findings from the Community Acquired Pneumonia Impact Study

**DOI:** 10.1186/1471-2318-10-1

**Published:** 2010-01-04

**Authors:** Eduardo Fernandez, Paul Krueger, Mark Loeb

**Affiliations:** 1 Department of Clinical Epidemiology and Biostatistics, McMaster University, Hamilton, Ontario, Canada; 2 Department of Family and Community Medicine, University of Toronto, Toronto, Ontario, Canada; 3 Departments of Pathology and Molecular Medicine, McMaster University, Hamilton, Ontario, Canada; 4 Michael DeGroote Institute for Infectious Diseases, McMaster University, Hamilton, Ontario, Canada

## Abstract

**Background:**

The purpose of this study was to identify predictors of health decline among older adults with clinically diagnosed community acquired pneumonia (CAP). It was hypothesized that older adults with CAP who had lower levels of social support would be more likely to report a decline in health.

**Methods:**

A telephone survey was used to collect detailed information from older adults about their experiences with CAP. A broader determinants of health framework was used to guide data collection. This was a community wide study with participants being recruited from all radiology clinics in one Ontario community.

**Results:**

The most important predictors of a health decline included: two symptoms (no energy; diaphoresis), two lifestyle variables (being very active; allowing people to smoke in their home), one quality of life variable (little difficulty in doing usual daily activities) and one social support variable (having siblings).

**Conclusions:**

A multiplicity of factors was found to be associated with a decline in health among older adults with clinically diagnosed CAP. These findings may be useful to physicians, family caregivers and others for screening older adults and providing interventions to help ensure positive health outcomes.

## Background

Morbidity in older adults is a growing problem in developed countries due to the increasing proportion of older adults in the population[1]. Although changes in physiology related to the aging process can explain some of the mechanisms involved in the development of greater morbidity and mortality in older adults, [2] there are a variety of other factors which are also important. For example, the availability of diagnostic and treatment facilities, social, economic and personal characteristics have all been shown to be associated with morbidity in this population. [3-6]

Respiratory infections such as influenza and pneumonia are common among older adults and are an important threat to the health of this population[7,8]. Although a variety of infectious agents (viruses and bacteria) are responsible for these infections, there are numerous risk factors which can predispose individuals to respiratory infections and a variety of prognostic factors which have been associated with the severity and recovery from these illnesses[9].

The Community Acquired Pneumonia Impact Study (CAPIS) was designed as a comprehensive community-based study to better understand the impact that community acquired pneumonia (CAP) has on older adults and their family caregivers. One of the study objectives of CAPIS was to identify predictors of health decline among older adults who developed CAP. It was hypothesized that older adults with CAP who had lower levels of social support (e.g. contact with family and friends) would be more likely to report a decline in health than those who reported higher levels of social support.

## Methods

CAPIS was a mixed methods, community-based study designed to identify the impact that CAP had on the lives of older adults and their family caregivers. This manuscript reports on findings from telephone interviews with older adults who were clinically diagnosed with CAP. Qualitative findings are reported elsewhere[10].

In order to get a broad picture of the impact that CAP has on older adults, data were collected on a wide variety of topics. Evans and Stoddard's model of the determinants of health[11] was used as a conceptual model to guide the collection of data for this project.

### Location

This study was conducted in Brant County, Ontario which includes the city of Brantford and the amalgamated County of Brantford. The population of Brant County at the time the data were collected was 118,485 with 14% of the population aged 65 years and older[10,12]. Brant County is a predominantly English speaking community with 86% of its population reporting English as first language. There were two community hospitals, eight radiology clinics and 80 family physicians.

### Participants

The study included all patients who were clinically diagnosed with CAP by their family physicians and who presented for chest x-rays to confirm the CAP diagnosis. The x-ray technicians at each of the eight radiology clinics were trained to recruit all potential candidates for the study (i.e. aged 60 or older presenting for a confirmatory chest x-ray) over a period of 15 months. A total of 195 older adults agreed to participate. Ethics approval was obtained from McMaster University and the Brant Community Health Care System.

### Survey Procedure

To avoid burdening older adults while they were ill, telephone interviews were scheduled four weeks after chest x-rays were taken. A trained interviewer telephoned each participant and collected detailed information including co-morbidities (e.g. allergies, diabetes, heart disease, cancer, liver disease), symptoms (e.g. shortness of breath, chills, sweats, pain during deep breaths, productive cough, energy level); lifestyle (e.g. activity level, smoking status, exposure to second hand smoke, alcohol consumption, nutrition, immunizations, spiritual values, pets); quality of life (using the SF8 to collect information on overall health, activity limitation because of health problems, difficulty doing usual daily activities because of physical health, amount of bodily pain, level of energy, limitations of social activities and daily activities due to personal or emotional problems); functional status (measured using the 10 item Barthel Index which includes grooming, dressing, feeding oneself, transferring from one's bed to a chair, bathing, toilet use, bladder control, bowel control, mobility, climbing stairs); instrumental activities of daily living scale (measured using the 8-item Lawton which includes items on meal preparation, mobility beyond short distances, shopping, phone calling, doing laundry, doing housework or handymen work, taking one's medication and money managing); social support (numbers and types of family, relatives, friends, distance to these contacts, frequency of contact, involvement in social and religious networks); and demographic characteristics.(age, gender, marital status, cultural background, language, employment history, living arrangements, level of education, household income, perceived level of social status). The reliability and validity of the above commonly used instruments can be found elsewhere [13-20].

### Dependent variable

One of the instruments we used to assess quality of life was the SF-8. The first question in this instrument asks participants to rate their overall health on a 6-point scale (1 = excellent, 2 = very good, 3 = good, 4 = fair, 5 = poor, 6 = very poor). The SF-8 is a well established measure of quality of life[13] and the single, overall self-rated health status question has been shown to be associated with morbidity and mortality in older adults [21-23]. In order to measure a decline in health resulting from CAP, we asked participants how they would have rated their overall health before they had CAP and then asked them to rate their overall health while they had CAP using the above response categories. A dichotomous outcome variable was created based on these responses, namely, those who did and those who did not report a decline in overall health.

### Predictor Variables

Participants were asked detailed information about their co-morbidities, lifestyle, quality of life, functional status, social support and demographic characteristics. Based on the literature and clinical experience, two investigators (EF and PK) independently reviewed the questionnaire for potential predictors to include in this analysis. In order to be selected as a potential predictor of the outcome variable, decline in health, each variable must have occurred prior to the participant recovering from CAP and then be selected by either of the two investigators.

### Analysis

Data from the telephone interviews were entered into and analyzed using SPSS 15 (SPSS, Inc., Chicago IL). Descriptive statistics were computed for all variables, including frequency counts and percentages for categorical variables, or means and standard deviations for continuous variables. For categorical variables, we used the chi-squared test, or when appropriate, Fisher's exact test to determine the significance of potential predictor variables. In addition, odds ratios (ORs) and 95% confidence intervals (CIs) are reported for each potential predictor of decline in health. T-tests were used to determine statistical significance for continuous variables.

Logistic regression analysis was used to identify the best predictors of decline in health. Only variables which had a statistically significant association in the above bivariate analyses were included in the logistic regression. Adjusted ORs and corresponding 95% CIs are reported for each variable in the final model. These ORs have been simultaneously adjusted for all other variables in the final logistic regression model. The goodness of fit of the logistic regression model was assessed using the rho-square statistic[24]. A rho-square value between 0.20 and 0.40 suggests a very good fit of the model. The Cox and Snell (R^2^) and Nagelkerke (R^2^) statistics are also reported. A probability level of <0.05 was used to determine statistical significance.

## Results

### Sample Characteristics

195 older adults with clinically diagnosed CAP completed telephone interviews of which: 61.5% were female; 65.2% were aged 70 or older (mean 72.8; standard deviation 6.8); 53.5% had completed high school; 62.8% were married or in common law; 93.7% had children; 75.3% had siblings; 77.2% were born in Canada; 76.2% owned their home; 58.8% rated their social status as 6 or higher on the MacArthur Scale of Subjective Social Status [25,26] (a socioeconomic status ladder with rungs from 1 to 10); and 66.9% had annual household incomes of $20,000 or more. On average, participants reported waiting 8.9 days (standard deviation 10.5) before seeking medical attention for their illness.

### Bivariate Analysis

A total of 88 variables were identified from the telephone interview as potential predictors of a decline in health. These variables were collapsed into the following categories: demographic characteristics (e.g. age, sex, education, income); co-morbidities (e.g. asthma, chronic bronchitis, diabetes, emphysema, heart disease); functional status (e.g. Barthel 10-item Activities of Daily Living (ADL) and Lawton 8-item Instrumental ADL scales); quality of life (e.g. SF-8 survey and CDC's 4-item health related quality of life measure); symptoms (e.g. chills, sweats, shortness of breath, no energy, sore throat, muscle aches); social support (e.g. children, siblings, other relatives, frequency of contact with friends or relatives), and lifestyle (e.g. immunizations, nutrition, pets, spiritual values, smoking, exposure to smoke, happiness).

Of the 88 potential predictor variables 17 were found to be statistically associated with a reported decline in health (Table 1). Of these 17, there was one demographic characteristic (gender); one comorbidity (heart disease); one functional status (Lawton IADL score); three quality of life (health problems that limited usual activities; difficulty doing daily activities because of physical health; amount of energy); five symptoms (sweats; shortness of breath; sore throat; no energy; headache); three social support (having siblings; having children; having pets); and three lifestyle variables (level of activity; household member smokes in the home; allowing people to smoke in the home).

**Table 1:** Variables statistically associated with a decline in self-reported health status

	Decline in Health Status			Unadjusted	
				
Variable	Yes Number (%)	No Number (%)	P value^1^	Odds Ratio	95% CI
Demographic Characteristics					

Gender (n = 192):					
Male	58 (77.3)	17 (22.7)		1.00	-
Female	103 (88.0)	14 (12.0)	0.049	2.16	(0.99, 4.69)

Co-morbidities					

Heart disease (n = 192):					
Yes	27 (69.2)	12 (30.8)		1.00	-
No	134 (87.6)	19 (12.4)	0.005	3.13	(1.36, 7.19)

Functional Status					

Lawton Instrumental Activities of Daily Living (IADL) score (n = 183):					
6 - 15	38 (76.0)	12 (24.0)		1.00	-
16^2^	117 (88.0)	16 (12.0)	0.045	2.31	(1.00, 5.32)

Quality of Life					

Before CAP, how much did health problems limit usual activities^3 ^(n = 192):					
Quite a lot/couldn't do	18 (66.7)	9 (33.3)		1.00	-
Not at all/very little/somewhat	143 (86.7)	22 (13.3)	0.020^4^	3.25	(1.30, 8.13)

Before CAP, how much difficulty doing usual daily activities because of physical health^3 ^(n = 192):					
Quite a lot/couldn't do	12 (60.0)	8 (40.0)		1.00	-
Not at all/very little/somewhat	149 (86.6)	23 (13.4)	0.006^4^	4.32	(1.59, 11.8)

Before CAP, how much energy did you have^3 ^(n = 192):					
A little/none	6 (46.2)	7 (53.8)		1.00	
Very much/quite a lot/some	155 (86.6)	24 (13.4)	0.001^4^	7.54	(2.33, 24.3)

Symptoms					

Sweats (n = 192):					
No	72 (37.5)	20 (10.4)		1.00	-
Yes	89 (46.4)	11 (16.1)	0.043	2.25	(1.01, 5.00)

Shortness of breath (n = 192):					
No	50 (73.5)	18 (26.5)		1.00	-
Yes	111 (89.5)	13 (10.5)	0.004	3.07	(1.40, 6.76)

Sore throat (n = 192):					
No	92 (78.6)	25 (21.4)		1.00	-
Yes	69 (92.0)	6 (8.0)	0.014	3.13	(1.22, 8.03)

No energy (n = 192):					
No	34 (69.4)	15 (30.6)		1.00	-
Yes	127 (88.8)	16 (11.2)	0.001	3.50	(1.57, 7.79)

Headache (n = 192):					
No	105 (80.2)	26 (19.8)		1.00	-
Yes	56 (91.8)	5 (8.2)	0.041	2.77	(1.01, 7.62)

Social Support					

Has brothers or sisters (n = 189):					
No	34 (72.3)	13 (27.7)		1.00	-
Yes	124 (87.3)	18 (12.7)	0.016	2.63	(1.17, 5.91)

Has children (n = 190):					
No	7 (58.3)	5 (41.7)		1.00	-
Yes	152 (85.4)	26 (14.6)	0.029^4^	4.18	(1.23, 14.2)

Has pets (n = 189):					
No	81 (78.6)	22 (21.4)		1.00	-
Yes	77 (89.5)	9 (10.5)	0.044	2.32	(1.01, 5.36)

Lifestyle					

Before CAP, how active (n = 188):					
Somewhat/not very/not at all	65 (76.5)	20 (23.5)		1.00	-
Very active	93 (90.3)	10 (9.7)	0.010	2.86	(1.26, 6.51)

Anyone in living in household regularly smokes inside the house (n = 190):					
No	123 (80.4)	30 (19.6)		1.00	-
Yes	36 (97.3)	1 (2.7)	0.013	8.78	(1.16, 66.6)

People allowed to smoke in the home (n = 190):					
No	100 (78.7)	27 (21.3)		1.00	-
Yes	59 (93.7)	4 (6.3)	0.009	3.98	(1.33, 11.9)

^1^Chi-square test

^2^A score of 16 indicates no help needed for any of the activities

^3^SF-8 questionnaire item

^4^Fisher exact test

### Multivariable Analysis

The 17 variables found to be statistically associated with a decline in health were then entered into a logistic regression analysis to determine the best predictors of a decline in health. The final logistic regression model included six variables (Table 2), two symptoms (no energy; sweats), two lifestyle (being very active; allowing people to smoke in their home), one quality of life (some to no difficulties doing usual daily activities) and one social support variable (having siblings). The final logistic regression model statistics are reported in Table 2.

**Table 2:** Final logistic regression model of the most important predictors of self-reported decline in health (n = 187^1^)

Predictors of Health Decline	Adjusted Odds Ratio^2^	95% Confidence Interval
Reported the symptom "no energy"		
No	1.00	-
Yes	5.06	(1.93, 12.70)

Before CAP, how much difficulty doing usual daily activities because of physical health^3^		
Quite a lot/couldn't do	1.00	-
Not at all/very little/somewhat	7.62	(2.09, 27.81)

People are allowed to smoke in the home		
No	1.00	-
Yes	4.70	(1.31, 16.86)

Has brothers or sisters		
No	1.00	-
Yes	3.30	(1.24, 8.81)

Before CAP, how active		
Somewhat/not very/not at all	1.00	-
Very active	3.04	(1.15, 8.06)

Reported the symptom "sweats"		
No	1.00	-
Yes	2.56	(1.00, 6.64)

Final Logistic Regression Model Statistics:

Rho-square = 0.27 (pseudo R^2^, values between 0.2 and 0.4 suggest a very good fit)

Cox & Snell R-square = .209; Nagelkerke R-square = .357 (i.e. between 20.9% and 35.7% of variance is explained by this model)

85.6% correctly classified

^1^Five of the 195 (2.6%) older adults had missing values for one or more of the variables included in the final model.

^2^Odds ratios for categorical variables represent comparisons with the referent group (OR = 1.00) after adjustment for all other variables in the model.

^3^SF-8 questionnaire item.

## Discussion

Data on health decline following CAP in the elderly is sparse. In this report, we identify six predictors (lack of energy, diaphoresis, being very active, allowing people to smoke in their home, little difficulty in doing usual daily activities, and having siblings) that are feasible to measure and can be used to identify health decline in this population.

Some of these predictors are in keeping with what may be reasonably expected. Reporting the symptom "no energy" for example, has face validity as a predictor of self-reported decline in health, particularly in a community-dwelling older adult population where many have responsibilities for their own and others' activities of daily living. A noted decline in energy, whether a result of fever, dehydration or other consequence of CAP would likely impact on their ability to function normally and would therefore effect their quality of life. Reporting diaphoresis may have been associated with subsequent dehydration or electrolyte imbalance[27].

Seniors reporting less difficulty doing usual daily activities because of physical health and those reporting to previously being very active were also more likely to report a decline in health. Those who were functioning at a high level before acquiring CAP may have been impacted more in their ability to function afterwards and therefore perceived a greater impact of CAP on their health because they became more reliant on others. Heidrich (2002) and Leinonen (1998) described how a decrease in the capacity to perform physical tasks is an important factor for older people when they assess their health and their functional performance in everyday life. It is clear that if an acute illness decreases physical health causing an impairment in these capacities, older people assign it a greater weight when assessing their health[28,29].

Our finding of an association between allowing people to smoke in the home and a reported decline in health is in keeping with previously described deleterious effects of smoking. Loeb(2009) and Jackson (2009) found that smoking played a role in the severity of pneumonia[30,31]. Almirall (2008) also stressed the role of passive smoking in the impact of pneumonia in the overall health condition of older individuals[32].

An unexpected finding was related to social support and decline in health. We initially hypothesized that older adults with CAP who had lower levels of social support would be more likely to report a decline in health. What we found was that those with siblings (a form of social support) were more likely to report a decline health. Older adults with siblings are likely to talk about their respiratory illnesses, which may result in a greater realization of the potential seriousness of CAP, which in turn could result in a reduced rating of their overall health. Unfortunately, however, we did not ask the participants why they rated their health lower so this explanation is speculative.

Strengths of this study include it being a community-based study that attempted to recruit all older adults over a 15 month time frame who were sent for a chest x-ray to confirm/rule out CAP. In addition, the selection of questions was guided by a broader determinants of health framework and resulted in the development and use of a comprehensive questionnaire which contained reliable and valid instruments to collect detailed information about a broad range of potential predictors.

There are several potential limitations of this study. The first limitation is that we only recruited older adults who went for chest x-rays. We therefore missed those who were treated for CAP by their physicians but were not sent for chest x-rays or who were sent but did not go. Sample size was also a limitation of this study, resulting in large confidence intervals. Since this study was done in only one relatively homogeneous community, the generalizability of the findings is another potential limitation. And finally, our definition of CAP was clinically diagnosed CAP versus x-ray confirmed CAP. The decision to use clinically diagnosed CAP versus x-ray confirmed CAP was based on there being no important differences in the characteristics or outcomes of those clinically diagnosed versus those with a positive chest x-ray; the fact that a large percentage of physicians do not send their patients for chest x-rays; and to increase the sample size for this analysis.

## Conclusions

In conclusion, we report a set of predictors that are easy to measure that could be used to screen for seniors who are more likely to have poorer health outcomes as a result of their CAP. The findings from this research show the value of taking a more in-depth determinants of health perspective when attempting to identify important predictors of health outcomes.

## Competing interests

The authors declare that they have no competing interests.

## Authors' contributions

EF had role in developing the research question, data analysis and interpretation, and was the lead writer of this manuscript. PK had a major role in the conception and design of the study, supervised all aspects of the study's implementation, had a role in data analysis and interpretation, contributed to the writing of the manuscript, provided editorial comments. ML contributed to the conception and study design, participated in data analysis and interpretation, contributed to the writing of the manuscript, and provided editorial comments. All authors read and approved the final manuscript.

## Pre-publication history

The pre-publication history for this paper can be accessed here:

http://www.biomedcentral.com/1471-2318/10/1/prepub
